# Understanding cervical cancer screening among lesbians: a national survey

**DOI:** 10.1186/1471-2458-13-442

**Published:** 2013-05-04

**Authors:** J Kathleen Tracy, Nicholas H Schluterman, Deborah R Greenberg

**Affiliations:** 1 Department of Epidemiology and Public Health, University of Maryland School of Medicine, 10 South Pine Street, MSTF 334-F, Baltimore, MD 21201, USA

**Keywords:** Homosexuality, Female, Papanicolaou test, Reproductive health, Health behavior

## Abstract

**Background:**

Lesbians have low rates of cervical cancer screening, even though they are at risk of developing the disease. The aim of this study was to examine cervical cancer screening behaviors in a national sample of lesbians.

**Methods:**

A standardized internet survey was sent to 3,000 self-identified lesbians to assess cervical cancer screening behaviors and barriers to screening. The sample consisted of 1,006 respondents.

**Results:**

Sixty-two percent of the weighted sample of respondents were routine screeners. Lack of a physician referral (17.5%) and lack of a physician (17.3%) were the most commonly-cited top reasons for lack of screening. Adjusting for age, education, relationship status, employments status, and insurance status, women who had disclosed their sexual orientation to their primary care physician (adjusted odds ratio [OR] 2.84 [95% confidence interval 1.82-4.45]) or gynecologist (OR 2.30 [1.33-3.96]) had greater odds of routine screening than those who did not. Those who knew that lack of Pap testing is a risk factor for cervical cancer were also more likely to be routine screeners (OR 1.95 [1.30-2.91]), although no association with screening was apparent for women who had more knowledge of general cervical cancer risk factors. Physician recommendation appeared to be a potent determinant of regular screening behavior. Routine screeners perceived more benefits and fewer barriers to screening, as well as higher susceptibility to cervical cancer.

**Conclusions:**

Some women who identify as lesbian are at a potentially elevated risk of cervical cancer because they are not routinely screened. Evidence-based interventions should be developed to address critical health beliefs that undermine participation in screening. Given the value placed on physician recommendation, patient-provider communication may serve as the optimal focus of effective intervention.

## Background

Cervical cancer is one of the most common reproductive cancers among women in the US [1-3]. The National Cancer Institute estimates that in 2011 roughly 12,710 cases of invasive cervical cancer were diagnosed in the United States, and approximately 4,290 women died from cervical cancer [4]. Cervical cancer incidence and mortality rates overall have decreased significantly during the last 50 years as a result of widespread cervical cancer screening with the Papanicolaou (Pap) test [5]. Lesbians comprise one subgroup of women who have underutilized the Pap test [6-8], and they may have unique reasons for not participating in routine cervical cancer screening [8,9]. Therefore, intervention techniques that are successful among heterosexual women may not be appropriate to use when targeting the lesbian population.

Cervical cancer screening prevents the occurrence of cervical cancer by allowing detection and treatment of pre-malignant lesions before invasive disease develops. Before an update to screening guidelines in early 2012 [10,11], the American Congress of Obstetricians and Gynecologists [10,11], the American Cancer Society [4], and the US Preventive Services Task Force [12] recommended that cervical cancer screening should: 1) begin no later than age 21 or within 3 years of the onset of sexual activity; 2) continue to at least age 65; and 3) occur at regular intervals—every 1–2 years up to age 30 and at least every 2–3 years thereafter—if the woman had no history of cervical abnormality.

The percentage of US women 18 and older reporting Pap smears within the last 3 years has been found to be 75-84% [13,14]. The published proportion of lesbians who have a recent Pap smear, however, is 44-57% [15-21], despite the fact that they too are at risk of developing cervical cancer [22-27].

Infection with oncogenic strains of human papillomavirus (HPV) is the biggest risk factor for cervical cancer, as the virus is present in 99% of cases worldwide [28]. Population-based prevalence rates of HPV infection among lesbians have not yet been published. Studies by Marrazzo, et al., note that 13-30% of US women who report having sex with women tested positive for HPV infection [22-26], compared to an overall HPV prevalence of 13-15% in North America [27], suggesting that woman-to-woman sexual transmission is possible. Other risk factors for cervical cancer, including smoking and obesity, may also occur more often among lesbians than in the general population [6]. Marrazzo and colleagues concluded that current recommendations for cervical cancer screening for lesbians should not differ from those for heterosexual women.

Most aspects of cervical cancer have not yet been well-studied in lesbians [9]. Potential barriers to routine cervical cancer screening in this group may include the perception that they are less susceptible to cervical cancer [29], experiences of discrimination and homophobia in the health care system, lack of health insurance, and fewer cues–such as contraceptive needs—to seek routine gynecologic care [6,8]. The relationships between lesbian patients and their health care providers (HCPs) may pose additional barriers: the HCPs may not have knowledge of disease risk in this population, or may fail to obtain a complete sexual history from lesbians, and lesbians may not be willing to disclose sexual orientation to HCPs [16,17,30-32].

In a small preliminary study [8], Tracy, et al., evaluated some of these barriers empirically in a sample of lesbians. Lesbians in that survey who did not routinely screen were less educated, but were not significantly less knowledgeable about cervical cancer risk factors than routine screeners. Non-routine screening was also associated with perceived discrimination in a variety of healthcare settings including hospitals, public health clinics, and community-based health clinics. Here, we extend the findings from that study by measuring factors specifically associated with cervical cancer screening in a large, nationally-based random sample of self-identified lesbians, using Internet survey methods.

## Methods

### Sample selection

The sample for this study was randomly selected from a Lesbian, Gay, and Bisexual (LGB) specialty panel tracked by Harris Interactive. The LGB specialty panel is a group of approximately 30,000 lesbian, gay, and bisexual individuals throughout the United States who have participated previously in at least one survey administered by Harris Interactive, reported sexual orientation of gay, lesbian, or bisexual, and consented to be re-contacted for potential recruitment for subsequent surveys. From this sampling frame, 4,422 women were randomly selected in proportion to age and race targets reflective of the presumed US lesbian population. Women were eligible for the study if they self-identified as gay or lesbian, were 21 to 70 years old, lived in the United States, and had no history of hysterectomy with removal of the cervix. The response rate was 35%, with 1,307 women responding to the survey and meeting inclusion criteria. Of these, 301 women declined to provide information about the length of time since their last Pap test, which precluded their categorization into a screening group; these women were excluded from further analysis. Participants were successfully recruited from all 50 states and the District of Columbia, with relatively equal representation from major geographic regions. The study was approved by the Institutional Review Board of the University of Maryland School Of Medicine.

### Measures

All study measures were collected via an interactive Internet-based survey designed by the first author in collaboration with Harris Interactive. Although the survey was constructed as one instrument, it included questions from the instruments described here.

#### Pap test screening and cervical cancer history

Standardized questions were used to ascertain the frequency of Pap screening, history of abnormal Pap screen results, and history of cervical cancer [13]. Data pertaining to frequency of Pap screening was used to categorize respondents into two screening groups [28]. Women were classified as routine screeners if they were 21–30 years old and reported a Pap test within the 12 months prior to participation in the study, or if they were 30 years or older and reported a Pap test within the 24 months prior to participation. All others were classified as non-routine screeners.

#### Knowledge of cervical cancer risk factors

Knowledge of risk factors for cervical cancer was measured using questions from the Harvard Center for Cancer Prevention (HCCP) “Your Disease Risk” publications [33] and American Cancer Society fact sheets for cervical cancer [34]. Age, smoking, sex at an early age, number of sexual partners, history of sexually transmitted diseases, use of condoms and diaphragms, number of births, and Pap test history were defined risk factors for cervical cancer based on evidence from the scientific literature; items asking about these factors were used as part of the HCCP assessment of risk factor knowledge. Fifteen yes/no questions about each risk factor were used to derive an overall knowledge score. Additionally, we analyzed the specific question asking women whether they knew that lack of Pap screening is a risk factor for developing cervical cancer. The score demonstrated adequate reliability, with a Cronbach’s alpha coefficient of 0.74 in this study for our derived knowledge scale score.

#### Perceived susceptibility, seriousness, barriers, and benefits of cervical cancer screening

Perceived susceptibility, seriousness, barriers, and benefits associated with cervical cancer screening were assessed using a modified version of Champion’s Health Belief Model Scale (CHBMS) [35,36] that has been described previously [8]. The modified scales demonstrated adequate internal consistency (Cronbach’s alphas: Susceptibility Scale = 0.90; Seriousness Scale = 0.87; Benefits Scale = .076; Barriers Scale = 0.81).

#### Perceived discrimination

Perceived discrimination was measured by two scales, yielding scores for everyday and general perceived discrimination. Everyday experiences of discrimination were assessed using the Perceived Discrimination scale from the Midlife Development Inventory (MIDI) designed and used in the Midlife in the US (MIDUS) study [37]. The Perceived Discrimination subscale is comprised of nine items that assess day-to-day experiences of discrimination. Items are rated on a 4-point scale (1=Often; 4=Never). This instrument demonstrated adequate psychometric properties (Cronbach’s alpha was 0.93). In addition, the number of perceived lifetime general discrimination events was assessed with an 11-question instrument asking how many times discrimination has occurred in the participant’s life (Cronbach’s alpha 0.74).

#### Disclosure of sexual orientation

Disclosure of sexual orientation (i.e., “outness”) to health care providers was assessed using items modeled from the Outness Inventory (OI) [38]. The OI is an 11-item self-reported measure designed to measure the extent to which an individual is open about her sexual orientation. For this study, women were asked to rate their level of outness to health care professionals, including their primary care physician and gynecologist; these ratings were then converted into a yes/no measure of outness to each HCP.

### Procedures

A letter of invitation to participate in the study was emailed to each member selected from the sampling frame. The invitation provided a brief description of the study and associated procedures and provided a hyperlink to the URL for the survey. The main page of the survey webpage presented respondents with an online version of the approved consent form, and respondents were asked to click a button to indicate willingness to participate. Once consent was obtained, participants were presented with a series of questions to determine their eligibility for participation. Those who were eligible proceeded to the full survey. Completion of the survey was anonymous. The survey was fielded from February-June 2010.

### Statistical analyses

Data were weighted based on demographic targets of the 21 to 70 year old lesbian or gay female population in the United States. Because no official US statistics are available for the lesbian population, demographic targets were internally derived by Harris Interactive. These targets were developed using estimates of the presumed demographic characteristics of lesbians in the general population, based on pooled data from online studies conducted among US adults regardless of sexual orientation. The demographic profile included age, education, race, region, income, and propensity score. The propensity score methods used by Harris Interactive then assigned a survey weight to each individual respondent in the sample. These propensity scores allowed respondents whose age, education, race, region, and income categories are underrepresented in online samples to contribute more weight during the analysis. As a result of this weighted sampling scheme, the data from the survey can be extrapolated onto the entire population of lesbians in the United States.

Data were examined for normality and completeness. All bivariate and multivariable analysis presented here have been weighted according to the survey weights provided by Harris Interactive. Bivariate analyses showed simple group differences between routine screeners and non-routine screeners on sociodemographic variables; group differences were assessed with Student’s t-tests (continuous variables) and Rao-Scott chi-square tests (categorical variables). Variables with more than two categories were dichotomized for the multivariable adjustment, as noted in Table 1. Multivariable logistic regression analyses were used to determine the independent association between cervical cancer screening behavior (routine screener v. non-routine screener) and several independent variables, adjusting for sociodemographic variables that were important predictors of screening behavior. A separate regression model was built for each of these primary independent variables: each of the aforementioned scales for knowledge, perceived susceptibility, seriousness, barriers, benefits, and discrimination, as well disclosure of sexual orientation to HCPs, HCP screening recommendation, and the specific question on the knowledge scale asking whether the women knew that lack of Pap testing is a risk factor for cervical cancer. For continuous independent variables, the odds ratios presented here represent the increased odds of regular screening per one-unit increase in the continuous variable; the regression model using number of general discrimination events, however, shows the increased odds of screening per 5-unit increase of discrimination events All statistical analyses were done using Stata 11 (Stata Corp., College Station, TX). Statistical significance was inferred at p<0.05.

**Table 1:** Sociodemographic and screening characteristics of survey sample

**Characteristics**	**Routine screeners**	**Non**-**routine screeners**	**Total**	**p**-**value**^**a**^
**Number (weighted % of total)**	644 (62.1%)	362 (37.9%)	1,006	
**Age (years, mean ± SD)**	44.7 ± 11.2	42.9 ± 12.3	44.0 ± 11.6	0.10
**Race (column %)**^**b**^				0.88
White	77.4%	76.3%	77.0%	
Black	7.2	8.7	7.8	
Other	15.4	14.9	15.2	
**College graduate (%)**	49.7	38.0	45.3	0.01
**Income (%)**				
≤ $24,999	17.2	25.4	20.3	0.02
$25,000 - $49,999	24.7	31.6	27.3	
$50,000 - $99,999	37.1	29.1	34.1	
$100,000 or greater	21.0	13.8	18.3	
**Employment status (%)**				0.04
Employed full time	54.3	41.5	49.4	
Employed part time	5.2	9.5	6.8	
Self Employed	7.6	13.1	9.7	
Unemployed	18.3	19.2	18.6	
Student	5.7	8.9	6.9	
Retired	9.0	7.8	8.5	
**Relationship status (%)**				0.01
Never Married	29.6	43.6	34.9	
Married/living together	58.9	46.9	54.3	
Separated/divorced	9.8	8.9	9.5	
Widowed	1.8	0.6	1.3	
**Census regions (%)**				0.21
East	26.2	18.5	23.3	
Midwest	17.5	21.9	19.2	
South	28.7	32.7	30.2	
West	27.5	27.0	27.3	
**Insurance type (%)**				<0.01
Covered by private plan	69.4	51.2	62.5	
Public assistance	16.6	18.4	17.3	
Other insurance	4.9	3.0	4.2	
No health insurance	9.1	27.5	16.1	
**Most recent Pap test (%)**				--
Less than 12 months	71.3	--	44.8	
12to 24 months	28.7	2.0	18.8	
>24 to 36 months	--	28.5	10.6	
>36 months	--	49.7	18.5	
Never	--	19.8	7.4	
**History of abnormal Pap (%)**	27.7	13.5	23.0	<0.01
**Previous CC diagnosis (%)**	1.7	0.7	1.3	0.39
**Family history of CC (%)**	9.1	3.1	6.8	<0.01

SD: standard deviation of individual participants; CC: cervical cancer.

All values and tests, except raw number of participants, are weighted according to survey weights.

^a^Rao-Scott chi-square tests, except age, which uses weighted Student’s *t*-test.

^b^All percentages are column percentages, except first row (Number).

## Results

### Participant characteristics

Participants were 1,006 women residing in the United States who met inclusion criteria and had sufficiently complete data to be categorized into a screening group. Table 1 shows sample characteristics and differences between routine and non-routine screeners. All participants were between the ages of 21 and 70 (weighted mean 44.0, SD 11.6). Majorities of the weighted sample were white (77.0%), and were married, in a civil union, or living with a partner (54.3%); pluralities were college graduates (45.3%), and were employed full-time (49.4%).

Routine screeners were more likely to have graduated college (p=0.01), more likely to be working full-time (p<0.01), more likely to be married or living with a partner (p=0.01), more likely to report an income over $50,000 (p<0.01), and more likely to be covered by any insurance (p<0.01) than non-routine screeners. Those who had recent screening were marginally older than non-screeners (p=0.10). Routine screeners and non-routine screeners did not differ substantially with respect to race or geographic region of residence.

**Figure 1:**
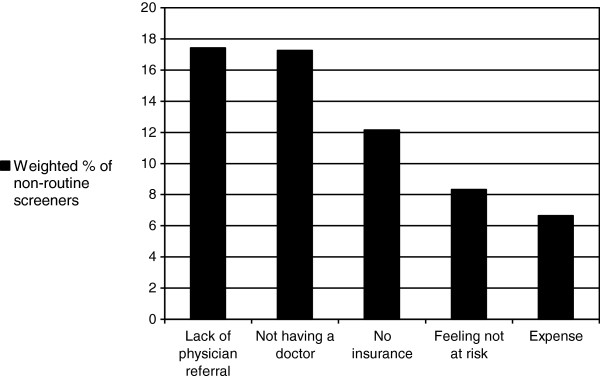
**Reasons given for not routinely getting cervical cancer screening among non**-**routine screeners.**

Among non-routine screeners, the most common top reasons given for not screening were lack of physician referral (17.5%) and not having a doctor (17.3%) (Figure 1). Lack of insurance was cited as the top reason for not screening for 12.2% of non-routinely screening respondents.

### Correlates to routine screening

Table 2 shows crude and adjusted relationships between cervical cancer screening behavior and key independent variables. Multivariable logistic regression models adjusted for age, education, relationship status, employment status, and insurance status. Knowledge of cervical cancer risk factors, perceived seriousness of cervical cancer, and perceived discrimination did not appear to have a substantial impact on screening behavior. However, the specific question on the knowledge scale pertaining to Pap testing as a risk factor was correlated with screening—those who answered this question correctly had nearly twice the adjusted odds of routine screening compared to those who did not answer correctly. “Outness” to HCPs was another important predictor of screening behavior, as women who had disclosed their sexual orientation to their HCPs had better screening behavior (adjusted odds ratio [OR] 2.84; 95% confidence interval [CI] 1.82-4.45), as did those who disclosed to their gynecologist (OR 2.30; 95% CI 1.33-3.96). Women whose health care professionals had recommended a Pap test were more likely to be routine screeners (unadjusted p<0.01), and had about twice the adjusted odds of routine screening. The perception that screening has benefits was associated with routine screening behavior (OR per unit increase 1.23; 95% CI 1.13-1.33), whereas those who perceived more barriers to screening had worse odds of regular screening (OR per unit increase 0.88; 95% CI 0.84-0.92).

**Table 2:** **Associations between independent variables and screening status** (**weighted**)

**Score**			**Mean ± SD, or %**			**Adjusted**^**c **^**odds ratio of being a routine screener (95% CI)**
	**Possible range**	**n with data**	**Routine screeners**	**Non-routine screeners**	**p-value**^**a**^	
**Number**			644	362		
**Knowledge of CC risk factors**	0-15	927	10.1 ± 2.5	10.0 ± 2.7	0.73	1.00 (0.92-1.09)
**Knowledge that lack of Pap test is CC risk factor**	Yes/no	984	67.7%	51.4%	<0.01^b^	1.95 (1.30-2.91)
**HMBS**-**susceptibility**	6-30	996	12.3 ± 4.7	11.5 ± 4.3	0.07	1.06 (1.01-1.11)
**HBMS**-**seriousness**	12-60	970	31.9 ± 9.0	31.2 ± 9.0	0.41	1.02 (0.99-1.04)
**HMBS**-**barriers**	9-45	975	18.0 ± 6.0	23.0 ± 5.5	<0.01	0.88 (0.84-0.92)
**HBMS**-**benefits**	4-20	980	16.0 ± 2.9	14.3 ± 3.0	<0.01	1.23 (1.13-1.33)
**Everyday discrimination (MIDI)**	9-45^e^	995	16.1 ± 5.9	16.2 ± 5.4	0.84	1.01 (0.97-1.05)
**Number of lifetime general discrimination events**	0-infinite	970	8.4 ± 33.8	7.0 ± 19.1	0.66	1.03 (0.99-1.07)^d^
**Disclosure of sexual orientation to HCPs – PCP**	Yes/no	910	72.0%	46.1%	<0.01^b^	2.84 (1.82-4.45)
**Disclosure of sexual orientation to HCPs – gynecologist**	Yes/no	688	78.3%	59.6%	<0.01^b^	2.30 (1.33-3.96)
**HCP recommended Pap**	Yes/no	1002	46.9%	28.9%	<0.01^b^	2.04 (1.32-3.15)

CI: confidence interval; CC: cervical cancer; SD: standard deviation of individual participants; HBMS: Health Belief Model Scale; HCP: health care provider.

^a^Student’s *t*-test with survey weights.

^b^Rao-Scott Chi Square test.

^c^Adjusted for age (continuous), education (college graduate and above vs. less than college graduate), relationship (living with a partner vs. not living with a partner), employment status (working full-time vs. not working full-time), and insurance status (having insurance vs. no insurance).

Odds ratios shown for continuous variables are per one-unit increase, except…

^d^Odds ratio per 5-unit increase.

^e^For MIDI only, higher values represent lower perceived levels.

## Discussion

Data from the current study indicate that less educated, lower income, and underinsured lesbians are less likely to participate in cervical cancer screening at recommended intervals. As in the previous study [8], we found that perceived barriers to cervical cancer screening were associated with lack of screening behavior.

Among the general population of women age 18 or older in the United States in 2008, about 75% of whites and 80% of blacks were routine screeners [14], somewhat higher than the 62% screening rate (fairly equitable between races) seen in the current study. The screening rate among lesbians in this study, however, was higher than the rate of 44-57% previously reported among US lesbians [15-21]. The difference in rates among lesbians may reflect part a time trend in the population, as many of the screening prevalence figures among lesbians were published more than a decade ago. In a more recent study published in 2008, Grindel et al. [39] showed that 75% of lesbians screened at least every two years, and an additional 13% screened every 3–5 years, bringing lesbians’ rates in line with that of the rest of the population. The results of the Grindel, et al., study must, however, be interpreted with a consciousness of their snowball sampling techniques that produced some quite unusual results, such as a finding that 80% of their overall sample reported having had an abnormal Pap test in their lifetime, which is far out of range of 20% expected in the general population [40]. Further study, using various population-based sampling techniques, is therefore needed to establish and monitor the screening rate among lesbians in the United States.

While previous studies have found that lesbians who are non-routine screeners are more likely to report discrimination due to sexual orientation than routine screeners in a variety of health care settings [6,16,17,30-32], this study found no compelling association between screening behavior and perceived everyday or lifetime discrimination in all settings. Non-routine screeners were, however, less likely than routine screeners to disclose their sexual orientation to their physician or gynecologist, and were less likely to report a physician recommendation. The reasons given by non-screeners echoed the importance of physician recommendation. With these observations taken together, we infer that a patient’s level of comfort and interaction with her HCP may be a more important determinant of screening behavior than her level of comfort with the health care system in general.

While some components of successful interventions among the general population may be appropriate to use when targeting lesbians, the data presented here imply that public health interventions may require specialized messages to improve cervical cancer screening coverage among this group. This study suggests that barriers to participation in routine cervical cancer screening among lesbians are directly related to the perceived benefits of screening, the perceived seriousness of cervical cancer, the perceived susceptibility to cervical cancer, and disclosure of sexual orientation to health care professionals. Interventions targeted to lesbians should therefore emphasize the benefits of screening and educate them of their susceptibility to cervical cancer, as well as offer strategies to overcome barriers.

The screening groups did differ in the specific knowledge that a lack of Pap testing can increase the risk of cervical cancer, but the groups appeared similar in general knowledge of risk factors. This indication that cervical screening among lesbians is related to knowledge of screening guidelines, but not knowledge of other risk factors, is consistent with the preliminary study [8]. Consequently, a screening education campaign directed to lesbians may be most effective if focused on screening guidelines.

Future investigations should explore the association between sexual behaviors, including sexual histories with male and female partners, and the perception of risk for acquiring HPV and cervical cancer. Incorporation of these types of questions into the evaluation of barriers to screening may provide insight into the decision-making processes related to cervical cancer screening, helping to disentangle the complex ways in which previous sexual behavior affects risk perception and subsequent screening behavior.

### Limitations

Our survey relied on self-report for categorizing women as lesbians and as routine or non-routine screeners. While the existing literature contains evidence that women tend to over-estimate their adherence with cervical cancer screening [41], others [42] have hinted that self-report offers a reasonable approximation of cervical cancer screening behavior. The validity of these measurements, therefore, should be subject to more scrutiny in future studies.

Although we surveyed a diverse group of women, representing all geographic areas of the US, the sample was self-selected, and probably over-sampled women with regular computer access. The survey weights assigned to participants made the results reflective of the best guess, based on the polling experience of Harris Interactive, of how the whole population of US lesbians probably looks.

## Conclusions

Lesbians are at risk of contracting HPV. However, this group participates in Pap screening exams at lower rates than those observed in the general population of women. In this survey, lesbians who did not screen for cervical cancer on the recommended schedule were less likely to disclose sexual orientation to a HCP and less likely to be knowledgeable of the benefits of Pap testing than those who do screen at recommended rates. Additionally, lesbians who were not routine screeners perceived more barriers and fewer benefits to screening than routine screeners, even after adjusting for age, education, and insurance status. Lack of a HCP recommendation was also a predictor of poor screening behavior. Public health interventions focused on increasing cervical cancer screening in lesbians should therefore be designed to educate this population about the risks of cervical cancer and the benefits of screening, suggest strategies to overcome barriers, and improve relationships between lesbians and their HCPs.

## Abbreviations

HPV: Human papillomavirus; HCP: Healthcare provider; LGB: Lesbian, Gay, and Bisexual; HCCP: Harvard Center for Cancer Prevention; CHBMS: Champion’s Health Belief Model Scale; HBMS: Health Belief Model Scale; MIDI: Midlife Development Inventory; MIDUS: Midlife in the US; OI: Outness Inventory; OR: Odds ratio; CI: Confidence interval.

## Competing interests

The authors declare that they have no competing interests.

## Authors’ contributions

KT conceived of the study objectives, designed the survey questionnaire, obtained funding and led the writing of the manuscript. NS and DG performed the statistical analyses and assisted with writing the manuscript. All authors approved of the final manuscript.

## Pre-publication history

The pre-publication history for this paper can be accessed here:

http://www.biomedcentral.com/1471-2458/13/442/prepub
